# Editorial: Management of patients with a failed kidney transplant: perspectives from transplant nephrologist, infectious disease, immunogenetics, oncology and transplant surgeons

**DOI:** 10.3389/fneph.2024.1455764

**Published:** 2024-08-15

**Authors:** Phuong-Thu T. Pham, Phuong-Chi T. Pham

**Affiliations:** ^1^ Department of Medicine, Nephrology Division, Kidney Transplant Program, David Geffen School of Medicine at University of California at Los Angeles (UCLA), Los Angeles, CA, United States; ^2^ Department of Medicine, Nephrology Division, Olive View-UCLA Medical Center, David Geffen School of Medicine at University of California at Los Angeles (UCLA), Los Angeles, CA, United States

**Keywords:** failed kidney transplant, immunosuppression continuation, immunosuppression weaning, dialysis modality, infection and mortality risk, allograft nephrectomy, allosensitization, re-allograft transplant

Patients who require dialysis reinitiation after a failed transplant have been shown to have higher mortality rates compared with those with a functioning allograft as well as transplant-naïve incident dialysis patients, particularly in the first several months or first year of dialysis reinitiation. Whether immunosuppression continuation vs. discontinuation, graft nephrectomy vs. no nephrectomy, or other factors define patient survival remain to be elucidated. In this Research Topic, we aim to address various challenging issues encountered in the management of patients with a failed transplant.

Kendrick provided a comprehensive review of the literature on various complex issues faced by transplant recipients with failing allografts. The author speculated that in addition to the chronic inflammatory state from the retained allograft, suboptimal care may contribute to the increased mortality risk in this group of patients. In an analysis using the national French Renal Epidemiology Information Network database, Mourad et al. showed similar survival among patients with failing transplants returning to dialysis compared with their transplant-naïve counterparts (1). It was postulated that better outcomes compared to other studies might be due to universal health care access in France. Retrospective studies designed to evaluate outcomes of transplant recipients with failing allograft managed in a dedicated low clearance transplant clinic (LCTC; eGFR < 20-30 ml/min) compared with those attending general transplant clinics showed better documentation of counseling and transplant work-up in center with LCTC compared to a center without such follow up. However, no difference in patient survival or control of CKD parameters were observed. The investigators speculated that the small number of patients in the study along with relatively short observation time may account for the lack of apparent impact in measures such as patient mortality (2, 3). Larger studies with long-term follow-up are needed.

The impact of dialysis modality on outcomes following failed transplants also deserves attention. Meta-analysis of small retrospective studies showed no increased risk of mortality, peritonitis, or technique failure in patients with failed transplants on peritoneal dialysis (PD) when compared to transplant naïve patients (4). Kendrick recommended that PD should be considered as a dialysis modality in the setting of a failed allograft. Compared to transplant naïve patients with end-stage kidney disease, relatively lower rates of PD use was seen among transplant recipients with failed allografts.

Similar to incipient transplant recipients, dialysis vintage has been shown to adversely affect survival of a subsequent transplant. Identifying candidates for repeat allograft transplant and early referral can maximize the chance of pre-emptive waitlisting and transplantation.

The review by Hickey et al. focuses on immunosuppression management in patients with failing allografts who remain potential re-transplant candidates. The authors speculated that a one-size-fits-all protocol is unlikely attainable given unique individual needs. However, the authors suggested that we can take lessons learned from transplant recipients with a functioning graft and apply them to those with a failed transplant. The Clinical Trial in Organ Transplantation (CTOT)-9 designed to study the effects of tacrolimus withdrawal at 6 months post-transplantation in non-sensitized living donor kidney transplant showed unacceptable rates of acute rejection and/or development of *de novo* DSA among those who underwent tacrolimus withdrawal compared with those randomized to remain on tacrolimus (5). This has led to the premature termination of the study. Similarly, several studies have demonstrated that calcineurin inhibitor (CNI) weaning/discontinuation in the setting of a failed transplant increased the risk for allosensitization. Taken together, these studies suggest that continued low dose CNI may be beneficial in avoiding allosensitization in re-transplant candidates with living donors.

Several retrospective cohort studies reported an increase in mortality and infections among patients with failed allografts maintained on immunosuppression. Yanagimoto-Ogawa et al. pointed out that in most studies, infection was commonly documented as a general event, rather than describing specific infectious organisms or etiology. The authors speculated that the greatest risk of infection may be due to catheter-related infections. In one multicenter prospective study consisting of 296 patients with a failed transplant who was recently initiated on dialysis, Knoll et al. demonstrated that prolonged use of immunosuppressants was not associated with a higher risk of hospitalization for infection compared with those who discontinued all immunosuppressants or continued prednisone only. Of interest, patients who continued immunosuppressants had a lower risk of death (adjusted HR 0.4) (6). The investigators emphasized the need for randomized controlled trials given the contrast in the results and concerns of bias when using observational data.

There has been no consensus on whether allograft nephrectomy (AN) should be performed in patients with a failed transplant due to controversial results reported in the literature. McDonald pointed out that to assess for benefit or harm from AN, it must be clear what outcome is being measured such as symptom mitigation, allosensitization, or survival following retransplant. As such, AN may be beneficial in patients with recurrent graft intolerance syndrome despite pulse steroids. The author reported that current evidence shows a trend toward increased anti-HLA antibody formation following AN and recommended that this factor should be considered in decision-making surrounding immunosuppression withdrawal. Studies evaluating the beneficial effect of AN on graft survival after retransplant are mainly inconclusive but suggest no overall survival benefit of subsequent graft from AN. The review by McDonald underscores the need to define specific outcome measures in AN decision-making given that the surgical procedure is not without morbidity and/or mortality risk.

Controversies over how to best manage patients with failed transplants remain due to the lack of randomized controlled trials. Nonetheless, this Research Topic provides important key take-home messages. Early referral for dialysis access to avoid use of central venous catheter and its associated infection risk should not be overlooked. The choice of dialysis modality should be based on patient preference. Determining whether patients are re-transplant candidates and inquiring about living donors are of paramount importance in guiding immunosuppression management. In addition, preemptive waitlisting and transplantation may improve outcomes. Studies evaluating optimal CNI drug levels to prevent allosensitization are needed. In patients awaiting re-transplantation, immunosurveillance using longitudinal solid-phase anti-HLA antibody testing or other biomarkers (e.g. cell-free DNA or gene expression profiling) can be invaluable. AN in the setting of graft intolerance syndrome refractory to steroid pulse is justifiable.
